# Prenatal diagnosis of Blepharo-Cheilo-Dontic syndrome: a case report

**DOI:** 10.1007/s00404-025-08268-0

**Published:** 2026-01-21

**Authors:** A. Rejaey, C. Berg, A. Reuss, I. Gottschalk

**Affiliations:** 1 MVZ Am Marienplatz 2, Witten, Germany; 2 Division of Prenatal Medicine, Gynecological Ultrasound and Fetal Surgery, Department of Obstetrics, Faculty of Medicine, University of Cologne, Cologne, Germany; 3 Praxis Central, Essen, Germany; 4 Schwerpunkt Pränatalmedizin, Gynäkologische Sonographie und Fetalchirurgie, Klinik und Poliklinik für Geburtsmedizin, Universitätsklinikum Köln, Kerpener Str. 34, 50931 Cologne, Germany

**Keywords:** Blepharo-Cheilo-Dontic syndrome, Prenatal diagnosis, Cleft

## Abstract

This case report describes the prenatal diagnosis of the extremely rare Blepharo-Cheilo-Dontic syndrome. After sonographic diagnosis of the bilateral cleft lip and palate and the persistent open eyelids, amniocentesis with subsequent molecular genetics confirmed the sonographically presumed de-novo mutation of the CDH1 gene and the Blepharo-Cheilo-Dontic Syndrome. After multidisciplinary counseling the patients termined the pregnancy.

## Take-home message

**Table:** 

Once a facial cleft is diagnosed prenatally, an accurate examination of the fetal eylids should be performed as the diagnosis of a cleft lip and palate with additional persistent open eyelids is strongly suspicious of a Blepharo-Cheilo-Dontic Syndrome which should be confirmed (or excluded) by genetic testing.	

**Fig. 1:**
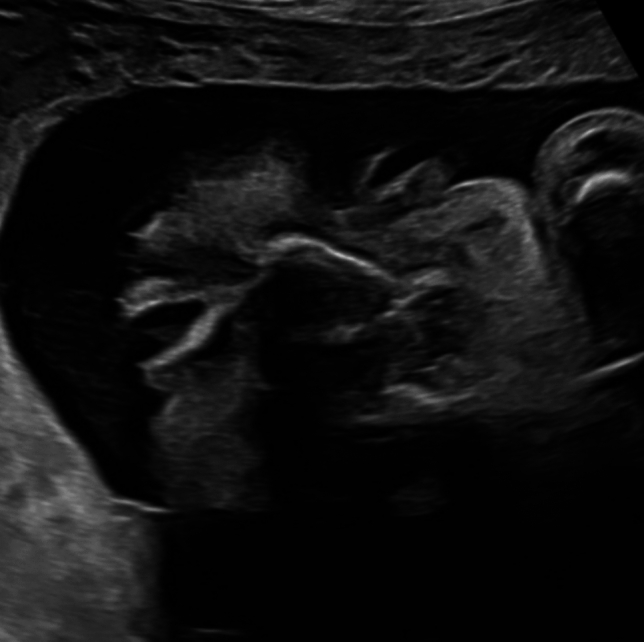
2D ultrasound in the frontal plane failed to demonstrate the closed eyelids

**Fig. 2:**
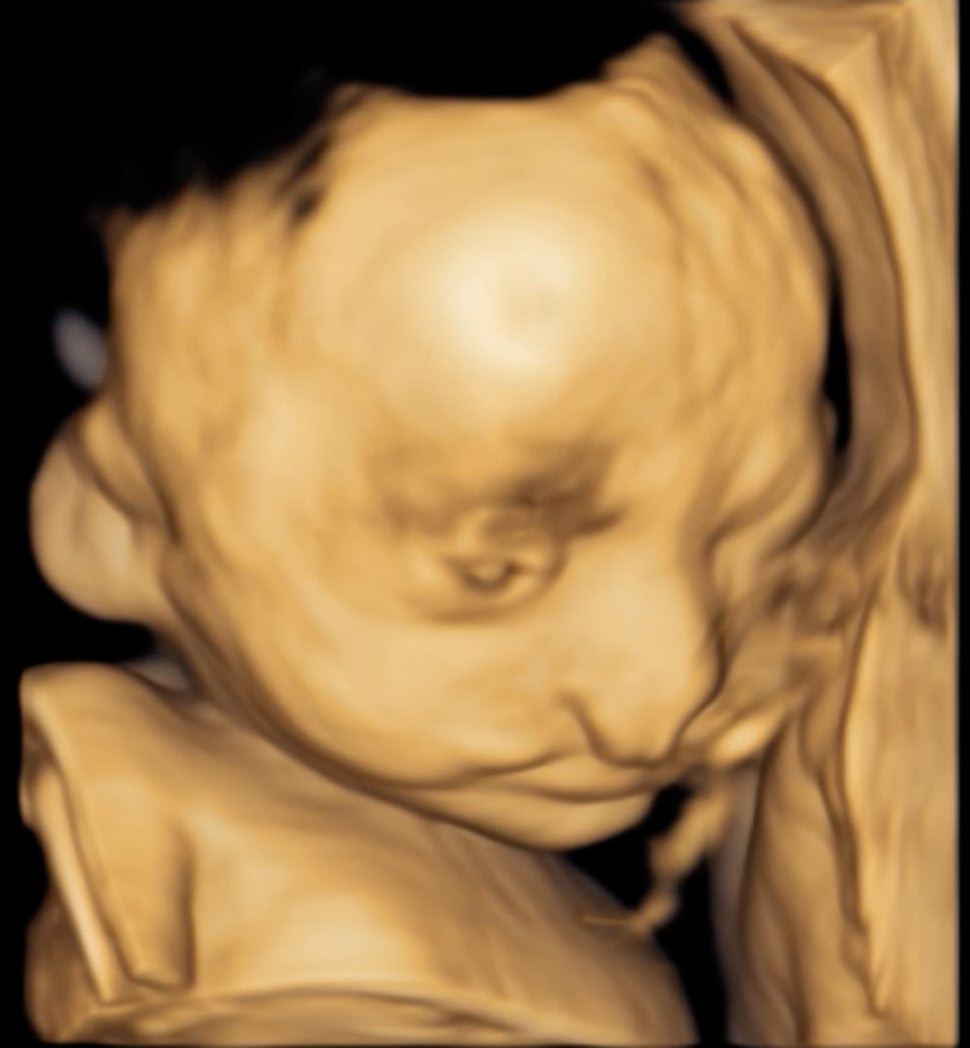
Open fetal eyelids in the 3D reconstruction

**Fig. 3:**
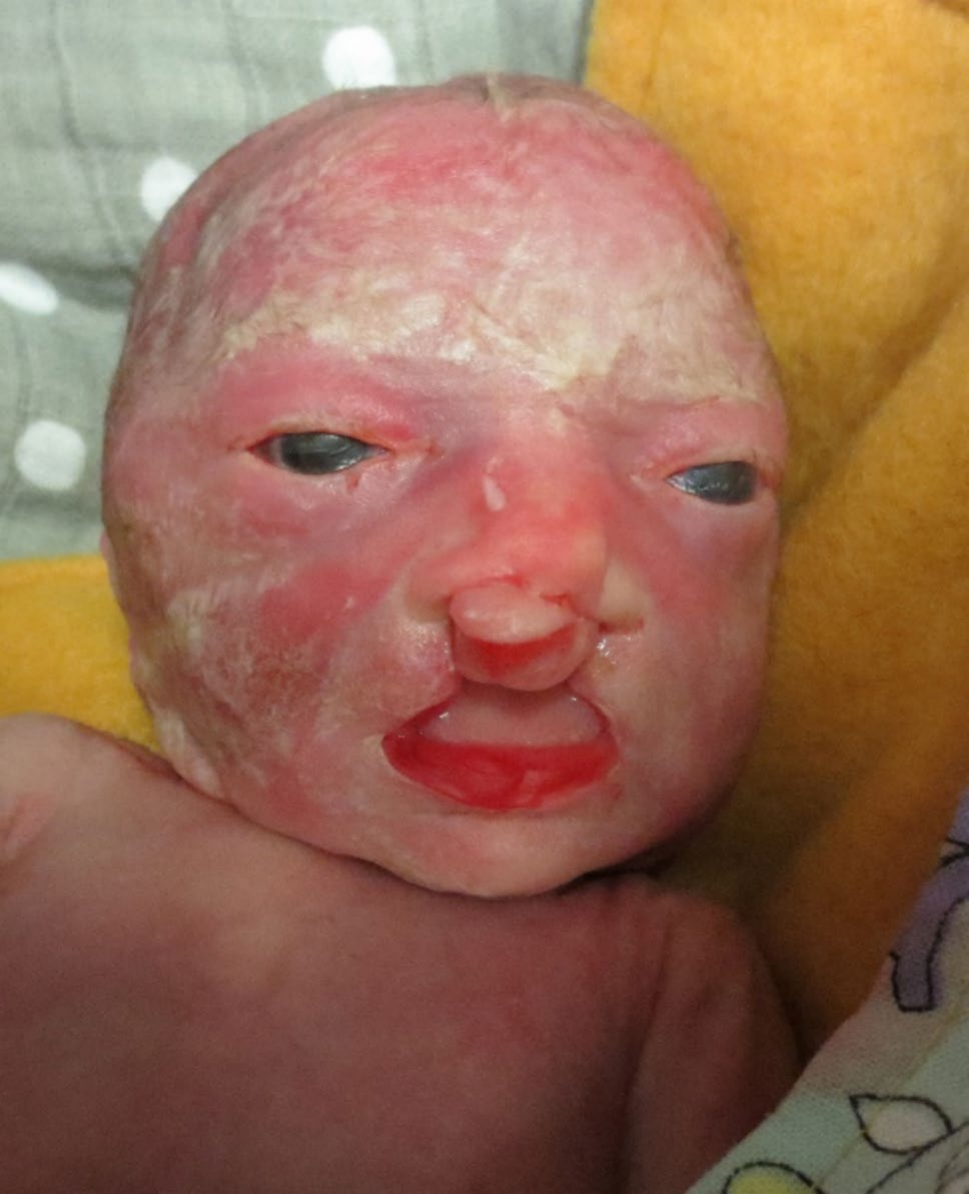
Postmortem findings demonstrating bilateral cleft lip and palate and open eyelids

A 37-year old patient in her second pregnancy presented at 23 weeks of gestation for an anomaly scan. Both parents are healthy without any known previous illness. The ultrasound examination revealed bilateral cleft lip and palate and 2D ultrasound failed to demonstrate the typically visible closed eyelids in the frontal plane (Fig. 1). 3D ultrasound confirmed that the fetus had persistently open eyelids (Fig. 2). The remaining fetal anatomy was normal. Based on these findings a presumptive diagnosis of a Blepharo-Cheilo-Dontic syndrome was made. Amniocentesis with subsequent molecular genetics confirmed a de-novo mutation of the CDH1 gene. After thorough genetic and ophthalmological counselling, the parents decided to terminate the pregnancy. The postnatal findings confirmed the prenatal diagnosis (Fig. 3).

Blepharo-Cheilo-Dontic syndrome is characterized by open eyelids and cleft lip and palate and typically presents with eyelid malformations such as euryblepharon, lagophthalmos, ectropion, and dental anomalies like hypodontia and conical tooth shape. Individuals with this syndrome generally have a normal IQ [1]. The feasibility of eyelid reconstruction depends on the extent of the remaining tissue. However, the risk of blindness is very high, along with other complications such as painful ingrown eyelashes [2]. Blepharo-Cheilo-Dontic syndrome is predominantly inherited in an autosomal dominant manner due to mutations in the CDH1 and CTNND1 genes, which encode cadherin, a calcium-dependent cell adhesion molecule crucial for cell mobility and epithelial proliferation [3].

## Data Availability

No datasets were generated or analysed during the current study.

## References

[1] Figueiredo J, Melo S, Carneiro P et al (2019) Clinical spectrum and pleiotropic nature of *CDH1* germline mutations. J Med Genet 56:199–208. 10.1136/jmedgenet-2018-105807 30661051 10.1136/jmedgenet-2018-105807 PMC6581119

[2] Yen MT, Lucci LM, Anderson RL (2001) Management of eyelid anomalies associated with blepharo-cheilo-dontic syndrome. Am J Ophthalmol 132:279–280. 10.1016/S0002-9394(01)00926-6 11476703 10.1016/s0002-9394(01)00926-6

[3] Zhao X, Li X, Sun W et al (2023) Case report: “Major fetal cardiac pathology associated with a novel CTNND1 mutation.” Front Pediatr 11:1180381. 10.3389/fped.2023.1180381 37274823 10.3389/fped.2023.1180381 PMC10235691

